# Fabrication of a 3D Nanomagnetic Circuit with Multi-Layered Materials for Applications in Spintronics

**DOI:** 10.3390/mi12080859

**Published:** 2021-07-22

**Authors:** Fanfan Meng, Claire Donnelly, Luka Skoric, Aurelio Hierro-Rodriguez, Jung-wei Liao, Amalio Fernández-Pacheco

**Affiliations:** 1 Cavendish Laboratory, University of Cambridge, Cambridge CB3 0HE, UK; cd691@cam.ac.uk (C.D.); ls604@cam.ac.uk (L.S.); j.liao@durhammagnetooptics.com (J.-w.L.); 2 SUPA, School of Physics and Astronomy, University of Glasgow, Glasgow G12 8QQ, UK; hierroaurelio@uniovi.es; 3 Departamento Fisica, Universidad de Oviedo, 33007 Oviedo, Spain

**Keywords:** 3D spintronics, 3D nanomagnetism, magnetotransport, 3D nanoprinting, magnetic thin films

## Abstract

Three-dimensional (3D) spintronic devices are attracting significant research interest due to their potential for both fundamental studies and computing applications. However, their implementations face great challenges regarding not only the fabrication of 3D nanomagnets with high quality materials, but also their integration into 2D microelectronic circuits. In this study, we developed a new fabrication process to facilitate the efficient integration of both non-planar 3D geometries and high-quality multi-layered magnetic materials to prototype 3D spintronic devices, as a first step to investigate new physical effects in such systems. Specifically, we exploited 3D nanoprinting, physical vapour deposition and lithographic techniques to realise a 3D nanomagnetic circuit based on a nanobridge geometry, coated with high quality Ta/CoFeB/Ta layers. The successful establishment of this 3D circuit was verified through magnetotransport measurements in combination with micromagnetic simulations and finite element modelling. This fabrication process provides new capabilities for the realisation of a greater variety of 3D nanomagnetic circuits, which will facilitate the understanding and exploitation of 3D spintronic systems.

## 1. Introduction

The field of spintronics explores the interaction of the magnetisation and spin current and finds a large range of applications in modern computing technologies such as sensing, data storage and logic, where most functionalities are implemented on 2D patterned planar single- or multi-layered structures [1]. The success in this field largely relies on the precise control of high quality materials in these structures, with in some cases even atomic-level control over the thickness and roughness of surfaces and interfaces being achieved [2]. This ability to control materials, especially in multilayered structures, has led to great discoveries such as Giant Magnetoresistance (GMR), Tunnelling Magnetoresistance (TMR) and Perpendicular Magnetic Anisotropy (PMA), resulting in the commercial realisation of scalable nonvolatile magnetic random-access memories [1]. Moreover, this fine control has made possible the introduction of the Dzyaloshinskii–Moriya interaction (DMI) [3] and Ruderman–Kittel–Kasuya–Yosida (RKKY) interaction into thin film magnetic systems, leading to the realisation of magnetic skyrmions and synthetic antiferromagnets [2,4]. However, as with CMOS devices, spintronics faces challenges in the continuous down-scaling of 2D devices and also the urgent demand for more energy-efficient devices and new functionalities [1].

The expansion of spintronics from 2D into 3D is considered one of the most promising solutions to this approaching limit due to the unique advantages offered by 3D structures [5,6]. For example, 3D structures could offer higher density and better device connectivity, with proposals for 3D racetrack devices offering high-density memory [7] and 3D interconnected memresistors for neuromorphic computing [8,9,10]. Moreover, the extra degree of freedom associated with the third dimension allows the manipulation of 3D geometrical effects such as curvature, chirality and topology and leads to new physics such as the observation of new magnetic textures [11] and curvature-induced effects [12,13], which can provide opportunities for new functionalities.

Creating 3D nanomagnets is, however, not trivial. So far, those used for experimental studies have been mainly achieved in three routes [6]. The first route uses a combination of 3D templates and chemical synthesis techniques such as electroplating, electroless deposition and atomic layer deposition. Anodised alumina matrices are the most common templates, producing arrays of high-aspect ratio cylindrical nanowires [11,14] that have led to the observation of new magnetic textures such as bloch point domain walls [11]. Truly 3D templates can be achieved via block-copolymers [15,16] and other self-assembly methods. In this way, 3D magnetic gyroid lattices have been realised, allowing for long-range ordered systems [16]. Although well suited to the fabrication of extended nanoscale systems, this fabrication route is limited in the choice of geometries and the control over the thickness of the ferromagnetic films grown via chemical synthesis methods, hence hindering the inclusion of functional interfaces [6]. The second route employs a 3D nano-printing technique [17], known as focused electron beam induced deposition (FEBID), which allows prototyping of individual complex 3D structures [18], with tens of nanometre resolution in polycrystalline or amorphous cobalt [19], iron or cobalt-iron alloys [20,21]. Artificial double-helices which provide controlled magnetic chirality have been realised using this method [22]. This route provides significantly more flexibility in the choice of geometry; however, it is limited by the range of materials and cannot be used to create high quality materials and interfaces directly [17]. The third route involves the use of physical vapour deposition (PVD) on top of previously patterned non-magnetic 3D scaffolds. These scaffolds can be created in various ways including two-photon optical lithography [10,23], self-assembly [24] and also FEBID [25]. This method has been used in the creation of a 3D nanomagnetic domain wall conduit [25] and also the realisation of a frustrated 3D nanowire lattice [10]. This route offers more flexibility in the choice of geometries and takes advantage of PVD which could offer high quality materials with more precise control. However, challenges are faced with regard to conformal and uniform deposition.

In addition to the fabrication of 3D nanomagnets, a second challenge to the realisation of 3D spintronic devices involves their integration into microelectronic circuits. Indeed, the experimental realisation of 3D nanomagnetic circuits has so far relied on fabrication-specific methods. For example, for the chemical deposition around 3D templates, magneto-transport studies have been achieved on cylindrical nanowires that are released from the template and electrically contacted via different micro-manipulation methods [14,26,27,28]. In these wires, discoveries such as fast current driven domain wall velocities [14] have recently been made. On the other hand, a main advantage of the direct writing of magnetic materials employing FEBID allows for one to directly deposit a 3D magnetic nanostructure on pre-patterned electrical contacts. In this way, the 3D geometrical effects on magneto-electrical signals have been studied in a 3D cobalt nanobridge system [29]. Until now, however, the realization of a 3D spintronic device based on PVD materials with a complex 3D geometry has not yet been realised.

In this work, we developed a new fabrication process for the realisation of 3D spintronic devices that combines 3D nanoprinting, PVD and lithography methods. This process not only allows us to take advantage of FEBID, which provides flexibility in defining geometries of the 3D scaffold, but also of PVD which allows for the incorporation of high quality materials. In particular, the use of PVD expands the choices of materials and makes the use of multi-layered materials in 3D nanomagnetic circuits possible. We demonstrate this new method through the creation of a 3D nanomagnetic circuit that integrates sputtered Ta/CoFeB/Ta multilayer onto a 3D nanobridge. The magnetic states of this 3D circuit are investigated by magnetotransport measurements aided by a combination of micromagnetic and finite element modelling. This method is not restricted to either a bridge geometry or Ta/CoFeB/Ta matireal, hence greatly expand the variety of 3D nanomagnetic circuits to be studied and pave paths to future studies of 3D spintronics effects, as well as the realisation of 3D spintronic technologies.

## 2. Materials and Methods

### 2.1. Fabrication of a 3D Nanomagnetic Circuit Using FEBID and PVD

**Figure 1:**
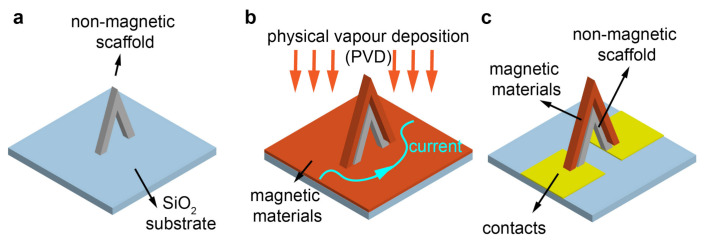
Creating a 3D nanomagnetic circuit using the combination of a scaffold and PVD. (**a**) Create a non-magnetic scaffold. (**b**) Incorporate magnetic materials using PVD, magnetic thin films deposited will shunt current from the 3D structure. (**c**) The ideal 3D nanomagnetic circuit created using ‘scaffold + PVD’ method.

Using physical vapour deposition on top of a non-magnetic scaffold (Figure 1a,b) is a reliable way to create 3D nanomagnets with complex geometries and high quality magnetic materials [10,25]. However, as shown in Figure 1b, one of the main obstacles preventing this method from being used in 3D spintronic devices is that with standard PVD techniques, the material is deposited everywhere on a substrate, meaning that without additional non-trivial fabrication steps, the current is shunted from the 3D structure and instead runs through the continuous thin film deposited around it.

Here, we create a 3D circuit based on a nanobridge coated with high quality magnetic materials (Figure 1c) by developing a fabrication process that overcomes the ‘current shunt’ constraint of the ‘scaffold + PVD’ method. A magnetic nanobridge represents one of the key building blocks in 3D spintronics. It is a basic element that can interconnect different parts of a 3D nanomagnetic circuit easily and is also capable of hosting domain walls and spin waves to serve as both a memory element and a logic gate. The nanobridge represents one of the building blocks for interconnecting the electrical and magnetic parts in 3D spintronics [30]. Here, the non-magnetic scaffold of the bridge is created by FEBID, exploiting the capability for mask-less fabrication of 3D structures with tens of nanometre spatial resolution. The 3D magnetic nanobridge is achieved by coating the scaffold with Ta/CoFeB/Ta deposited with UHV DC magnetron sputtering. CoFeB, a workhorse of spintronic devices [31], is chosen due to its high spin polarisation and moderate saturation magnetization which are essential for reducing the current density required for current-induced magnetisation switching [32]. The integration of CoFeB onto a 3D nanobridge thus represents the first realisation of a 3D spintronic device with these material properties.

**Figure 2:**
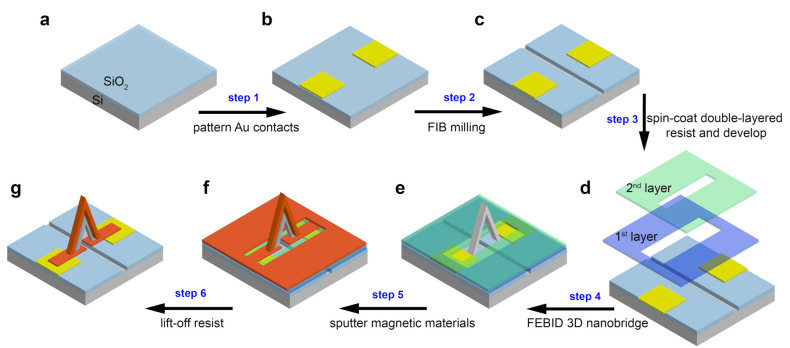
Fabrication process. (**a**) Prepare a clean silicon substrate with 300 nm thermally oxidised silicon dioxide. (**b**) Pattern Au contacts using optical-lithography and sputtering. (**c**) Mill a trench between two contacts using focused ion beam. (**d**) Spin-coat and pattern the double-layered resist. (**e**) FEBID 3D Pt-C scaffold (**f**) Sputter multilayered materials. (**g**) Lift-off resist.

**Figure A1:**
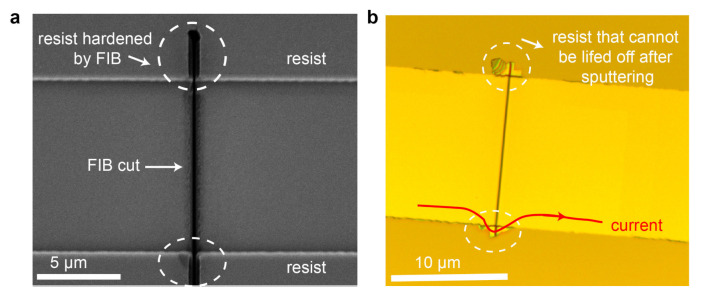
The consequence of performing FIB milling after the deposition of resist. (**a**) The SEM image taken after FIB milling with resist around. (**b**) The optical microscopy image taken after metallisation: the region irradiated by FIB cannot be lift off.

**Figure A2:**
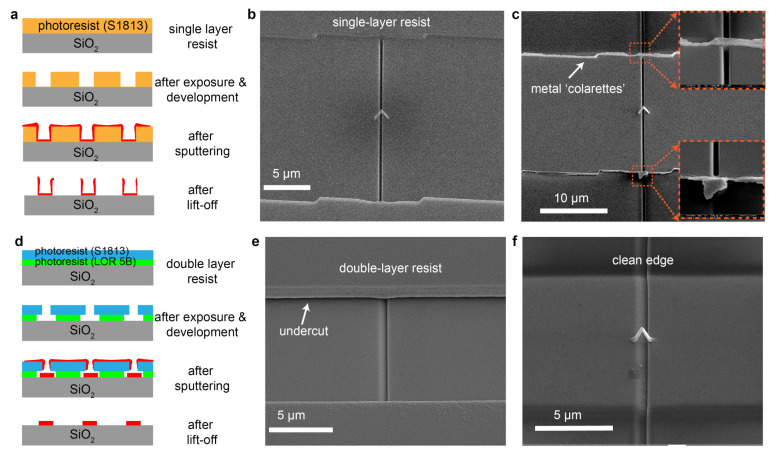
Comparison between using single and double layer resist. (**a**) Schematics of using single layer resist. (**b**,**c**) SEM images taken before and after metallisation when using single layer resist. (**d**) Schematics of using double layer resist. (**e**,**f**) SEM images taken before and after metallisation when using double layer resist.

To overcome the shunting effect, in additional to sputtering thin films on top of a non-magnetic scaffold created by FEBID, we make use of patterned resist to limit the area of the deposited thin films, as well as a milled trench under the 3D structure to ensure the current only flows through the top of the 3D bridge. A description of the overall fabrication process is given in Figure 2. At the beginning of the process, Au contact pads are patterned on a clean silicon dioxide substrate using optical-lithography and sputtering (Figure 2a,b). Next, a trench is milled by focused ion beam (FIB) between the two contact pads (Figure 2c), designed to prevent the formation of a continuous film under the bridge. FIB milling is a key step of this fabrication process, and it needs to be completed before the use of resist. This is due to the fact that if the resist is exposed to the ion beam, it will harden and therefore cannot be removed in the subsequent lift off process, leading to metallic lift off edges spanning the trench, which provide paths for the current to shunt (details are shown in Figure A1 in Appendix A). Milling the trench beforehand also avoids inadvertent gallium ion implantation, which can lead to the deterioration of the magnetic material [33]. The width and depth of the trench should be large enough to prevent that a continous film is formed within the trench. In our case, for a total 30 nm thick materials to be supttered, we milled a trech of width = 0.85 μm and nominal depth = 500 nm, which is sufficient for this purpose, as demonstrated by the electrical insulation test shown later in Section 2.2. The double-layer resist is then patterned using optical lithography to open a narrow window on top of the trench and contacts (Figure 2d). The use of double-layer resist is essential for a clean lift-off after sputtering which is a non-directional deposition method. This cannot be achieved using single-layer resist without the assistance of ultrasonic bath which will damage the 3D structure. To facilitate the lift-off process without using ultrasonic bath, we used a warm SVC 14 solution which is a relatively strong resist stripper. A detailed comparison of the effects of single and double-layered resist are given in Figure A2 in Appendix A.

**Figure 3:**
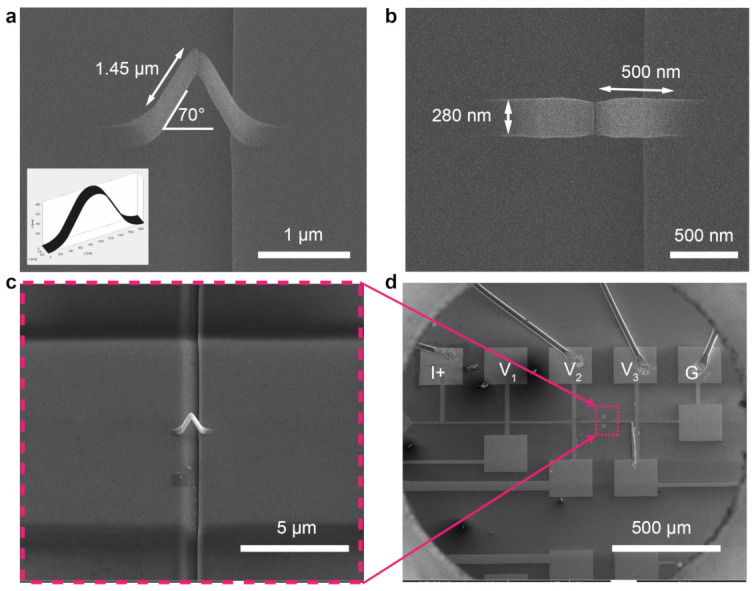
Creating a 3D nanomagnetic circuit. (**a**) SEM image of the printed Pt-C scaffold (stage tilt at 30${}^{\circ }$). Inset is the CAD design of the bridge. (**b**) Top view of the printed Pt-C bridge. (**c**) Side view of the printed Pt-C bridge (stage tilt at 30${}^{\circ }$). (**d**) Top view of the printed 3D nanomagnetic circuits with electrical connections shown.

Within the window opened, a non-magnetic 3D bridge is deposited across the trench (Figure 2e) via FEBID combined with a recent CAD-implementation, which allows the deposition of complex nanostructures directly from standard 3D computer aided design (CAD) files [18]. A 3D Pt-C bridge (CAD design shown in Figure 3a) is deposited using a non-magnetic precursor (CH${}_{3}$)${}_{3}$Pt(C${}_{p}$CH${}_{3}$) with an accelerating voltage and beam current of 30 kV and 21 pA, respectively. A layer of Ta/Pt (2/10 nm) is sputtered on top of the sample prior to the FEBID deposition to alleviate charging effects during the deposition. The front and top view of the fabricated bridge scaffold (Figure 3a,b) show that the legs of the fabricated bridge are 1.45 μm long, 280 nm wide, forming an angle of 70${}^{\circ }$ with the substrate. From the top view, we observe a reduced width and thickness with respect to the CAD file at the middle part of the bridge where the two legs connect. This is due to the very shallow angle of that central part, which leads to a reduction in the number of secondary electrons generated and hence slows down the growth speed [34]. Further optimization would be required for a better match with the design. However, this structure works well for the purpose of this paper, focused on the demonstration of the integration of multilayered material on a 3D nanostructure.

Finally, a thin film of Ta/CoFeB/Ta (2/20/2 nm) is deposited with DC magnetron sputtering (Figure 2f) and the resist is lifted off, leaving the 3D scaffold coated with the magnetic thin film and connected to the electrical contacts by the 2D magnetic tracks (Figure 2g). The 3D nanomagnetic circuit achieved after lift off is shown in Figure 3c,d, where we see a 3D bridge spanning over a FIB milled trench and is connected into the electrical circuit.

### 2.2. Electrical Insulation Verification of the FIB Milled Trench and the ‘Non-Conducting’ 3D Scaffold

The functionality of this magnetic 3D nanobridge relies on two main factors: first, that the shunting of current by thin film is prevented, and second, that the 3D scaffold is non-conductive and the current only flows through the 3D magnetic layer. We determine whether this is the case by comparing the resistance measured across the fabricated magnetic nanobridge (Figure 3c,d) which is around 30 kΩ, with two designed control structures.

**Figure 4:**
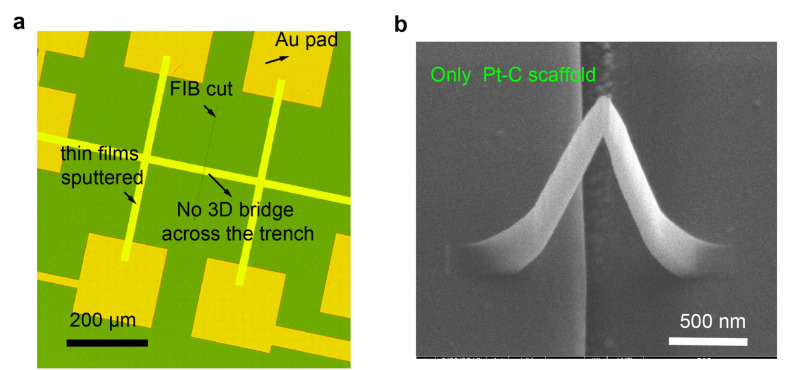
Electrical insulation verification of the trench and Pt-C scaffold. (**a**) An optical microscopy image of the FIB test device (without 3D bridge). (**b**) The SEM images of the Pt-C scaffold device (without magnetic multilayered materials).

First, we test whether the FIB milled trench successfully prevented shunting of the current by repeating the fabrication process (Figure 2) without depositing the 3D bridge (skip Step 4). This test sample is shown in Figure 4a and the two-probe resistance measured using a probe station with a Keithely 4200 SCS semiconductor system across the trench is 31.6 ± 1.6 GΩ. This is six orders of magnitude higher than the resistance measured across the magnetic bridge, hence we conclude that the FIB cut provides enough insulation.

We then check that the resistivity of the 3D scaffold is much higher than the magnetic thin films. Another device is fabricated using the same process, but without sputtering the thin film (skip steps 5 and 6) so that we can measure the resistance of the Pt-C bridge scaffold (Figure 4b).The resistance of the Pt-C scaffold is 3 GΩ which is also significantly larger than resistance measured across the bridge with magnetic thin film on top (five orders of magnitude higher). The corresponding resistivity of the scaffold is 1.5 × 10${}^{9}$μΩcm, which is in the range of reported values (10${}^{6}$ to 10${}^{12}$μΩcm) for Pt-C structures grown using (CH${}_{3}$)${}_{3}$Pt(C${}_{p}$CH${}_{3}$) precursor [35]. More details about the electrical verification tests on these two test devices can be found in Appendix B.

## 3. Results

**Figure 5:**
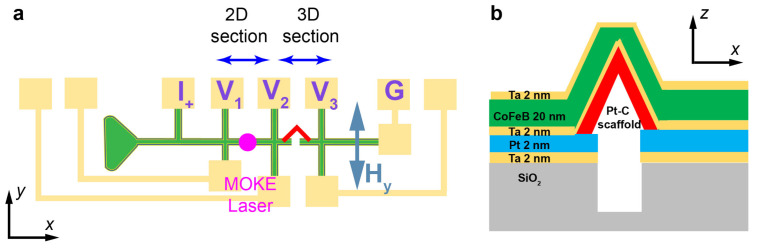
(**a**) The schematic that shows the MOKE and MT measurement positions. (**b**) The schematic of the cross-section of the 3D device.

Following the validation of this fabrication process, we next consider the magneto-transport (MT) results obtained from the 3D nanomagnetic circuit. The measurement configuration is shown in Figure 5a. A standard 4-terminal technique was used for the MT measurements, with an AC current of constant magnitude of 2.5 μA supplied between I${}_{+}$ and G. Hence, the current flows along the long-axis of both the 2D magnetic track and the 3D bridge and an in-plane magnetic field $H_{y}$ of up to 12 mT is applied perpendicular to the current direction. We measure not only the voltage drop across the 3D bridge section (V2–V3) but also the 2D track section (V1–V2). By comparing the two measurements, we can determine the magnetic and magneto-transport properties of the 3D nanobridge. Both transverse and longitudinal Magneto-Optic Kerr Effect (MOKE) measurements are also taken for the 2D track section with a laser diameter of 5 μm to obtain complementary information for the magnetization reversal process of this region.

Before we study the results of the MT measurements, we first consider the magnetoelectrical signals that are probed in this measurement geometry, and what we can learn from the data. In this geometry, we measure the longitudinal voltage along the current direction and hence probe the anisotropic magnetoresistance (AMR) which results in a change in the longitudinal electric field. This change depends on the angle between the current and magnetisation [36]:

(1) $$E=\rho_{⊥}+\left(\rho_{\|}-\rho_{⊥}\right){\left[m\cdot J\right]}^{2},$$

where E is the electric field, J is the current density vector, m is a unit vector in the magnetisation direction, $\rho_{\|}$ and $\rho_{⊥}$ are the resistivities for J parallel and perpendicular to m and $\frac{\rho_{\|}-\rho_{⊥}}{\rho_{⊥}}$ is the AMR ratio.

To understand the MT measurements, we need to access both m and J. The different resistivity of the layers in the multi-layered material (Figure 5b) and in combination with the 3D geometry bring complexity to the current distribution in the system. Thus, to understand the experimental MT results we employ a combination of micromagnetic simulations and a current simulation based on finite element method to take both the magnetic configuration and current distribution into account.

### 3.1. 2D Track Section: MOKE and Magnetotransport Results and Simulations

**Figure 6:**
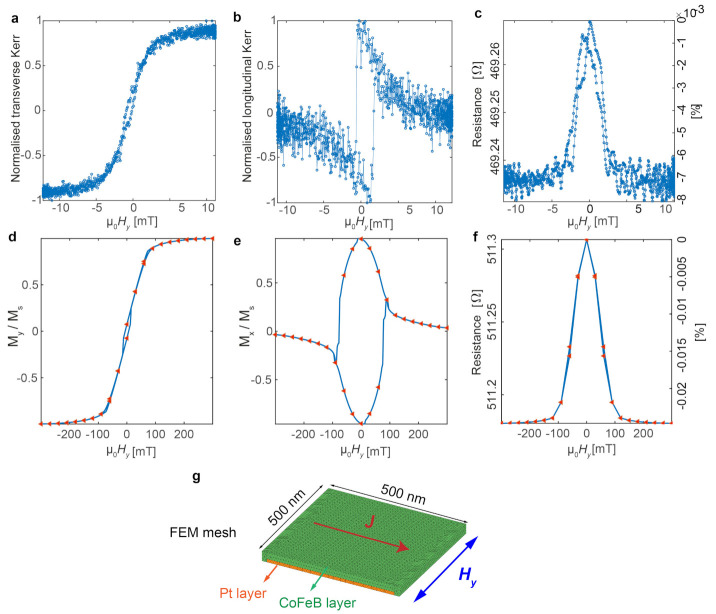
2D track section: MOKE and magnetotransport results and simulations for $H_{y}$ applied perpendicular to the long axis. (**a**) Normalised transverse Kerr (proportional to $M_{y}$). Error = ±0.03 (**b**) Normalised longitudinal Kerr (proportional to $M_{y}$). Error = ±0.12 (**c**) Magnetoresistance measured across the 2D track section (V2–V1). Error = ±2 mΩ (**d**,**e**) $M_{y}$/$M_{s}$ and $M_{x}$/$M_{s}$ results from Mumax3 simulation. (**f**) The simulated MT result based on the combination of Mumax3 and FEM simulation. (**g**) The FEM mesh used for the 2D track section.

We first consider the 2D track section, without the nanobridge. For $H_{y}$ applied perpendicular to the long axis of the 2D track, we measured both the transverse Kerr signal ($M_{y}$/$M_{s}$) and the longitudinal Kerr signal ($M_{x}$/$M_{s}$) that provide access to the components of the magnetisation perpendicular and parallel to the current, respectively (Figure 6a,b). As the field is applied along the hard axis, typical quasi-reversible $M_{y}$/$M_{s}$ loops with a small coercive field of ≈0.5 mT are observed. On the other hand, the *x* component of magnetization (parallel to the easy axis) has a maximum at around 0 T and exhibits a progressive decrease on the approach to saturation, except for the sudden magnetization reversal jumps at $\mu_{0}$$H_{c}$≈ 2 mT. These results indicate that in this area the magnetization reversal in the *y* direction occurs by coherent rotation for a wide range of fields with the additional jumps observed in $M_{x}$ consistent with a small *H*-misalignment with the hard axis [37]. The corresponding MT signal during reversal is shown in Figure 6c. The peak we see at 0 T is consistent with the $M_{x}$ measured since AMR is proportional to the $M_{x}^{2}$ (from Equation (1)), given that CoFeB has a positive AMR ratio [37].

To simulate the MT result, we first run micromagnetic simulations to obtain the magnetisation switching profile. The actual volume of magnetic material in the 2D section is 250 μm × 15 μm × 20 nm (aspect ratio: L/W=15), which is too large for our micromagnetic simulations. Hence, we use a down-scaled model of size 1024 nm × 256 nm × 20 nm (L/W=4). The shape anisotropy ($K_{s}=\frac{1}{2}M_{s}^{2}N$, where $M_{s}$ is the saturation magnetisation and *N* is the demagnetising factor along the long axis direction) of the real 2D track and the down-scaled model are 760 J/m${}^{3}$ and 9000 J/m${}^{3}$, respectively. To compensate for this change of shape anisotropy associated with the changed aspect ratio, we include a uniaxial anisotropy term of Ku1 = 5000 J/m${}^{3}$ which is of the same order of magnitude with the change and gave the best agreement between simulations and experiments. Typical simulation parameters for CoFeB are used as follows: mesh size = 5 nm, $M_{s}$ = 8 × 10${}^{5}$A/m and $A_{ex}$ = 0.9 × 10${}^{-11}$J/m [37,38]. Using this model, we obtain the $M_{y}$ and $M_{x}$ profiles as shown in Figure 6d and e that qualitatively agree well with the experiment. This agreement confirms our understanding that the 2D track under $H_{y}$ is switched mainly via coherent rotation.

After obtaining the magnetic profile from the micromagnetic simulation, we simulate the corresponding MT signal by solving the electric potential across the film using a FEM method with mesh shown in Figure 6g which takes into account both the Pt (deposited before FEBID to reduce charging effects) and CoFeB layers. For the Pt layer, we use a constant resistivity $\rho_{Pt}$ = 30 μΩcm [39] and for the CoFeB layer, we use a magnetisation dependent resistivity tensor $\rho_{CoFeB}\left(m\right)$ which can be obtained from Equation (1), using the magnetization profile simulated, $\rho_{⊥}$ = 230 μΩcm [37] and an AMR ratio of 0.15% [37] (details of the setup of the FEM model are given in Appendix C). Again to save computational time, the size of the FEM model was decreased (500 nm × 500 nm, L/W=1). To obtain a resistance that is comparable to the experimental results of the 2D track section, we scale up the quantitative FEM simulation results by a factor of 15 which accounts for the difference in aspect ratio between the real structure and the model. The simulation results are shown in (Figure 6f). The simulated resistance is around 510 Ω which agrees well with the data (≈469 Ω) and the peak we see at 0 T is also reproduced. The maximum percentage drops seen in the simulation and experiment are 20 × 10${}^{-3}\%$ and 8 × 10${}^{-3}\%$, respectively. The drop in the simulation is about 2 times larger which may be due to the fact that we ignore the Ta layers in our FEM model. These layers will lead to a further reduction of the percentage change. Other factors such as the differences of AMR ratio between CoFeB alloys with different stoichiometry or microstructure may also play a role. The good agreement between the simulation and experiments confirms our understanding of the switching process of the 2D section and the AMR contribution to the MT signal.

### 3.2. 3D Bridge Section: Magnetotransport Results and Simulations

**Figure 7:**
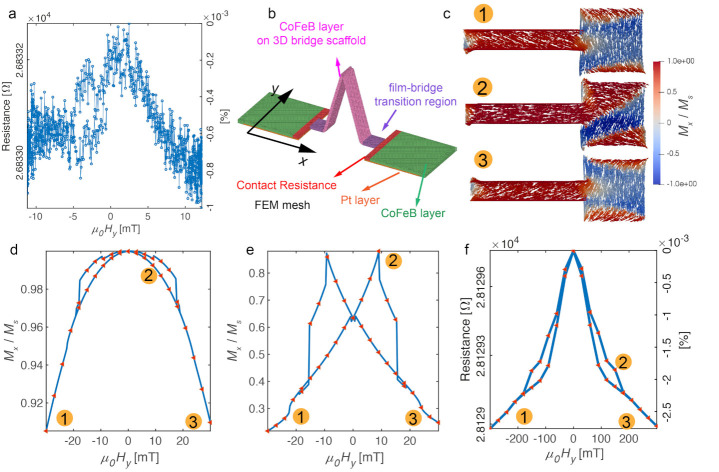
3D bridge section: magnetotransport results and simulations. (**a**) Resistance measured across the 3D section (V3–V2). (**b**) FEM model of the 3D section that consists: 2D track, 3D bridge and 2D to 3D transition region. (**c**) Three magnetization states from the micromagnetic simulation for the combination of the 3D bridge and the transition region ($M_{x}$/$M_{s}$ is plotted). (**d**) Simulation result of *$M_{x}$/$M_{s}$* for the nanowire section. (**e**) Simulation result of *$M_{x}$/$M_{s}$* for the transition section. (**f**) Simulated MT result based on the combination of Mumax3 and FEM simulation.

Having successfully reproduced the 2D track results, we now turn to the 3D magnetic nanobridge. This section consists of a 2D thin film track which is similar to the one studied in the previous section, but with a FIB milled trench in the middle and a 3D nanobridge which essentially acts as a constriction [40] in between two planar microwires. The MOKE signal from a 3D nanostructure such as the bridge is very challenging to obtain with respect to a planar system and requires recently-developed techniques that exploit dark-field effects [41]. Furthermore, in our case, diffuse reflection originating from the FIB milled trench with a curved profile makes this approach impractical. The MT measurement of this section is shown in Figure 7a. By comparing with the one obtained from the 2D track section (Figure 6c), we see that by including a 3D bridge into the circuit, the hysteresis loop becomes asymmetric with non-saturating resistance at high fields and distinctive jumps observed around −5 mT. The non-saturating resistance suggests that the system is not fully switched by the applied magnetic field. From the analysis in the previous section, we know the 2D track section is saturated by these fields. Therefore, we infer that the 3D nanobridge is not fully switched due to the shape anisotropy. Furthermore, the jumps in the hysteresis loop are due to the formation of a domain wall between these two sections.

To assist our understanding of the MT results of the 3D section, we create an FEM model (Figure 7b) that considers contributions from three parts: the 3D nanobridge (pink region), the 2D track (green) and the transition region between the 2D track and 3D bridge (purple). To obtain the corresponding resistivity tensor ρ(m) needed for each part of the FEM model, we again employ micromagnetic simulations. Specifically, for the 2D track section, the magnetisation profile that was confirmed in the previous section is used. For the 3D bridge and transition region is modelled together as a nanowire with a square pad attached at the end, as shown in Figure 7c. The same mesh size, $M_{s}$ and $A_{ex}$ are used for CoFeB as in the previous section [37,38]. The AMR signal we studied in this experiment measures how much magnetisation deviates from the current direction. With a 12 mT in-plane field applied in the y-direction, the magnetisation remains in the CoFeB film due to shape anisotropy, which is also the current plane. Thus, the measured AMR only depends on the x-component of the magnetisation. Figure 7c shows three magnetisation states at different fields, and the full simulated $M_{x}$/$M_{s}$ hysteresis loops with a maximum $H_{y}$ of 30 mT applied are plotted for the nanowire and pad section separately as shown in Figure 7d,e. From Figure 7d, we see the normalised $M_{x}$ of the nanowire sections changes from 0.9 to 1, indicating that the wire has not been switched. On the other hand, from state 1 and state 3 shown in Figure 7c, the transition area has mostly switched and the maximum $M_{x}$ appears when two 90 degree domain walls are pinned in this region. By substituting these magnetisation profiles into the corresponding resistivity tensors, we obtain the magnetotransport simulation shown in Figure 7f. This simulation result qualitatively reproduces the features from the data suggesting that the main drop in the signal is due to the switching of the 2D film with low coercive fields. Furthermore, the non-saturating AMR is due to the wire not being fully switched and the small additional drop at ≈5 mT is due to the DW pinning in the transition area. The asymmetrical loop seen in the results could be potentially due to local stress-induced anisotropy caused by the 3D bridge during the deposition of thin films [42,43].

**Figure 8:**
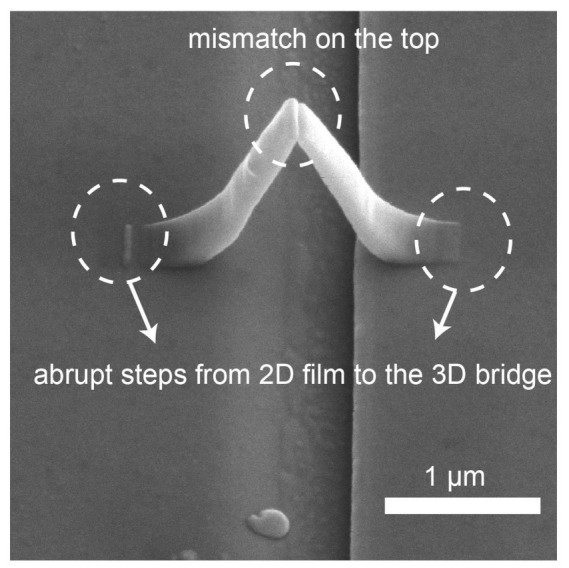
The SEM image of the 3D nanomagnet created where the imperfections caused by Pt-C are shown in white circles.

We note that the measured resistance is about 28 kΩ and the expected resistance of these sputtered materials is around 1 kΩ. The measured resistance is significantly larger which is likely to be due to imperfections from the Pt-C scaffold where a mismatch in the top region of the bridge is observed. In additional to the mismatch, there are sudden changes in surface height at the transition region between the 2D film and 3D bridge as shown in Figure 8. These abrupt steps lead to non-uniform step coverage and likely result in defects [44] in the sputtered thin films and hence a large contact resistance [45]. We included a constant contact resistance of 27 kΩ in the FEM model to compensate this discrepancy in the measured and simulated resistance. The maximum percentage change due to AMR from the simulation is 2.5 × 10${}^{-3}\%$ and is 1 × 10${}^{-3}\%$ from the experiment. The percentage change simulated is the same order of magnitude with the experiment, which indicates that this unexpected large resistance is not from a magnetic source, and also that the 2D micromagnetic model is adequate to capture the essence of the switching process.

## 4. Conclusions

We developed a new fabrication process which is based on FEBID and sputtering for prototyping 3D spintronic devices with complex geometries and multi-layered materials. Specifically, FIB milling and double-layered resist have been employed to overcome the ‘current shunt’ difficulties introduced by the PVD methods. A 3D nanobridge circuit with Ta/CoFeB/Ta materials on top has been created using this method and its magnetoelectrical response has been studied. By comparing the results from both the 2D and 3D sections aided by micromagnetic and FEM simulations, we verified that the 3D nanomagnet has been connected into the 2D circuits successfully and observed domain wall pinning at the 2D and 3D transition area. The principle of this method can be extended to other materials and geometries leading to a wide range of opportunities for the exploitation of the interplay between 3D geometry and magnetotransport.

## Figures and Tables

**Figure A3:**
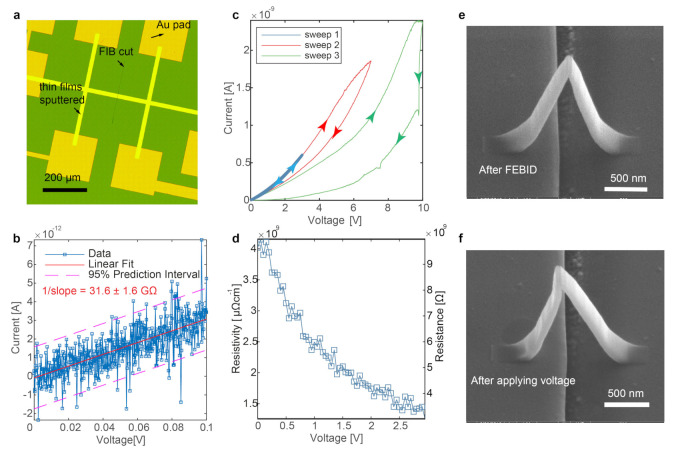
Electrical insulation verification of the trench and Pt-C scaffold. (**a**) An optical microscopy image of the FIB test device (without 3D bridge). (**b**) I–V curve measured across the FIB milled trench. (**c**) I–V curves of 3 voltage sweeps applied across the Pt-C scaffold. (**d**) Differential resistivity calculated from the first sweep. (**e**,**f**) SEM images taken before and after applying a 10V voltage sweep.

## Data Availability

In order to comply with EPSRC policy, the metadata associated to this publication can be found at https://doi.org/10.17863/CAM.71024.

## Abbreviations

The following abbreviations are used in this manuscript:

2D	Two dimensional
3D	Three dimensional
GMR	Giant Magnetoresistance
TMR	Tunnelling Magnetoresistance
PMA	Perpendicular Magnetic Anisotropy
DMI	Dzyaloshinskii–Moriya Interaction
RKKY	Ruderman–Kittel–Kasuya–Yosida Interaction
CMOS	Complementary Metal-Oxide-Semiconductor
FEBID	Focused Electron Beam Induced Deposition
PVD	Physical Vapor Deposition
UHV	Ultra High Vacuum
CAD	Computer Aided Design
DC	Direct Current
FIB	Focused Ion Beam
SEM	Scanning Electron Microscopy
MOKE	Magneto-Optic Kerr Effect
MT	Magneto-Transport
AMR	Anisotropic Magnetoresistance
FEM	Finite Element Method

## Appendix A. More Details about the Fabrication Process

### Appendix A.1. The Consequence of Performing FIB Milling after Having Resist

Figure A1 shows the consequence of perform FIB milling after depositing the resist. The region of resist that has been irradiated by the ion beam will be hardened and cannot be lifted off after metallisation. These metallic lift off edges will bridge the trench and provide paths for the current to shunt. In this work, milling parameters are set as the following: accelerating voltage = 30 kV, beam current = 0.28 nA, beam diameter overlap = 50%, dwell time = 1 μs.

### Appendix A.2. The Comparison between Using Single or Double Layered Resist

With single layer resist used, as the sputtering technique employed here for incorporating multilayered materials is a non-directional deposition method, we observe the formation of significant lift off edges (Figure A2a–c) which will shunt the current. Double layer resist can lead to the formation of an undercut due to the top layer resist (S1813) being sensitive to light, while the under layer only being dissolved through direct contact with the developer through the opening of the top layer (Figure A2d). The degree of the undercut only depends on the dissolution time, and is not related to laser exposure dose at all. This undercut (Figure A2d,e) ensures the deposition of a discontinuous film formed after sputtering and as shown in Figure A2f, results in a clean edge without any shunting of the current.

## Appendix B. Electrical Insulation Verification of the Trench and Pt-C Scaffold

To test the insulation of the FIB cut, the two probe I-V curve across the trench is measured as shown in Figure A3b using a probe station with a Keithely 4200 SCS semiconductor system. The resistance of 30 GΩ is calculated by measuring the slope.

To check that the resistivity of the 3D scaffold is much higher than magnetic thin films, another device is fabricated using the same process, but without sputtering thin films (skip step 5 and 6) so that we can measure the resistance of the Pt-C bridge scaffold. Three sweeps of voltage have been applied, 0-3-0 V, 0-7-0 V and 0-10-0 V respectively (Figure A3c). Since from the second sweep, we see permanent change of the bridge, so we measure the differential resistivity for the bridge from the first sweep as shown in Figure A3d. The decreasing resistivity with increasing voltage is the behaviour expected, as the Pt-C scaffold is essentially Pt inclusions in a carbonaceous matrix [35]. The minimum resistivity calculated is around 1.5 × 10${}^{9}$μΩcm, which is in the range of reported values (10${}^{6}$ to 10${}^{12}$μΩcm) for Pt-C structures grown using (CH${}_{3}$)${}_{3}$Pt(C${}_{p}$CH${}_{3}$) precursor [35] and is also significant larger than the magnetic materials we will use. Deformations of the bridge is observed clearly from the comparison of SEM images take before and after the electrical tests (Figure A3e,f) and it also proves that current flows through the bridge and hence demonstrates the effectiveness of the FIB trench.

## Appendix C. FEM Simulation of AMR

The influence of AMR effect on the electrical current can be described as a magnetisation-dependent resistivity tensor **ρ**(**m**) which is reformulated from Equation (1).

(A1) E=ρ(m)J,

(A2) $$\rho \left(m\right)=\left(\begin{array}{ccc} \rho_{⊥}+\left(\rho_{\|}-\rho_{⊥}\right)m_{x}^{2} & \left(\rho_{\|}-\rho_{⊥}\right)m_{x}m_{y} & \left(\rho_{\|}-\rho_{⊥}\right)m_{x}m_{z}\\ \left(\rho_{\|}-\rho_{⊥}\right)m_{x}m_{y} & \rho_{⊥}+\left(\rho_{\|}-\rho_{⊥}\right)m_{y}^{2} & \left(\rho_{\|}-\rho_{⊥}\right)m_{y}m_{z}\\ \left(\rho_{\|}-\rho_{⊥}\right)m_{x}m_{z} & \left(\rho_{\|}-\rho_{⊥}\right)m_{y}m_{z} & \rho_{⊥}+\left(\rho_{\|}-\rho_{⊥}\right)m_{z}^{2} \end{array}\right),$$

where $m_{x}$, $m_{y}$ and $m_{z}$ are the *x*, *y* and *z* component of the magnetisation vector **m**.

The conductivity tensor is then obtained by inverting the resistivity tensor, $\sigma =\rho^{-1}$. For each section of the FEM model and each magnetisation state at each applied field, a unique conductivity tensor is calculated from the Mumax3 and assigned to the corresponding sections of the model. The electric potential *u* at the side contacts is then computed by solving the partial differential equation, ∇·[−σ(∇u)]=0, using finite element method inplemented with a CAD-design-based finite element mesh.
